# The *Potorous* CPD Photolyase Rescues a Cryptochrome-Deficient Mammalian Circadian Clock

**DOI:** 10.1371/journal.pone.0023447

**Published:** 2011-08-16

**Authors:** Inês Chaves, Romana M. Nijman, Magdalena A. Biernat, Monika I. Bajek, Karl Brand, António Carvalho da Silva, Shoko Saito, Kazuhiro Yagita, André P. M. Eker, Gijsbertus T. J. van der Horst

**Affiliations:** 1 Department of Genetics, Center for Biomedical Genetics, Erasmus University Medical Center, Rotterdam, The Netherlands; 2 Laboratory of Virology, Wageningen University, Wageningen, The Netherlands; 3 Department of Neuroscience and Cell Biology, Kyoto Prefectural University of Medicine, Kyoto, Japan; Vanderbilt University, United States of America

## Abstract

Despite the sequence and structural conservation between cryptochromes and photolyases, members of the cryptochrome/photolyase (flavo)protein family, their functions are divergent. Whereas photolyases are DNA repair enzymes that use visible light to lesion-specifically remove UV-induced DNA damage, cryptochromes act as photoreceptors and circadian clock proteins. To address the functional diversity of cryptochromes and photolyases, we investigated the effect of ectopically expressed *Arabidopsis thaliana* (6-4)PP photolyase and *Potorous tridactylus* CPD-photolyase (close and distant relatives of mammalian cryptochromes, respectively), on the performance of the mammalian cryptochromes in the mammalian circadian clock. Using photolyase transgenic mice, we show that *Potorous* CPD-photolyase affects the clock by shortening the period of behavioral rhythms. Furthermore, constitutively expressed CPD-photolyase is shown to reduce the amplitude of circadian oscillations in cultured cells and to inhibit CLOCK/BMAL1 driven transcription by interacting with CLOCK. Importantly, we show that *Potorous* CPD-photolyase can restore the molecular oscillator in the liver of (clock-deficient) *Cry1/Cry2* double knockout mice. These data demonstrate that a photolyase can act as a true cryptochrome. These findings shed new light on the importance of the core structure of mammalian cryptochromes in relation to its function in the circadian clock and contribute to our further understanding of the evolution of the cryptochrome/photolyase protein family.

## Introduction

Life is subject to the 24-hour rotation cycle of the earth, which imposes rhythmic changes in light and temperature conditions. In order to anticipate these environmental changes, most organisms have developed a circadian clock with a period of approximately 24 hours that allows them to adjust behavior, physiology and metabolism to the momentum of the day. To keep pace with the day/night cycle, this internal clock needs to be reset every day, using light (the most predictable environmental cue) as the strongest Zeitgeber (German for “time giver” or synchronizer). The mammalian master clock is located in the suprachiasmatic nuclei (SCN) of the hypothalamus, and receives light-induced signals from the retina via the retino-hypothalamic tract [1]. In turn, this master clock sends humoral and neuronal signals that synchronize peripheral oscillators, located in virtually every cell or tissue [2]–[5].

The mammalian cryptochrome proteins (CRY1 and CRY2) belong to the cryptochrome/photolyase family (CPF) of flavoproteins and were initially identified as homologues of photolyase [6], [7]. In view of their strong resemblance to plant cryptochrome proteins, which act as blue light photoreceptors, the mammalian CRY proteins were hypothesized to act as photoreceptors for resetting of the circadian clock [6], [8]. Unexpectedly however, inactivation of the *Cry1* and *Cry2* genes in the mouse was shown to shorten or lengthen the period length of the circadian clock respectively, whereas in the absence of both genes circadian rhythmicity was completely lost [9]–[11]. This observation, together with the finding that the *Cry* genes encode the most potent inhibitors of the circadian transcription activator CLOCK/BMAL1 [12], positioned the mammalian CRY proteins at the heart of the circadian core oscillator.

The mammalian circadian clock consists of a molecular oscillator, composed of a set of clock genes that act in transcription-translation-based feedback loops. The CLOCK/BMAL1 heterodimer activates transcription of the *Period* (*Per1*, *Per2*) and *Cryptochrome* (*Cry1*, *Cry2*) clock genes through E-box elements in their promoter. Following synthesis, the PER and CRY proteins will gradually accumulate in the nucleus and ultimately repress CLOCK/BMAL1, and thereby transcription of their own gene [13]–[15]. A second loop is formed by REV-ERBα, which cyclically inhibits RORα-driven transcription of the *Bmal1* gene [16]–[19]. Adding to this transcription/translation feedback loop mechanism is a network of post-translational modifications of clock proteins (phosphorylation, (de)acetylation, sumoylation and ubiquitylation) that fine-tune the period length of the circadian oscillator and confer robustness and persistence to the molecular clock [20]–[27].

Photolyases, the other members of the CPF, are DNA repair enzymes that use visible light to lesion-specifically remove ultraviolet light-induced cyclobutane pyrimidine dimers (CPDs) or (6-4) pyrimidine-pyrimidone photoproducts ((6-4)PPs) from the DNA in a reaction called photoreactivation [28], [29]. Placental mammals have lost photolyase genes during evolution and solely rely on nucleotide excision repair for removal of CPDs and (6-4)PPs [30]. Nevertheless, when expressed in the mouse, CPD and (6-4)PP photolyases rapidly remove these UV-induced lesions in a light-dependent manner and protect the animal from sunburn, mutation induction, and skin cancer development [31]–[33].

**Figure 1:**
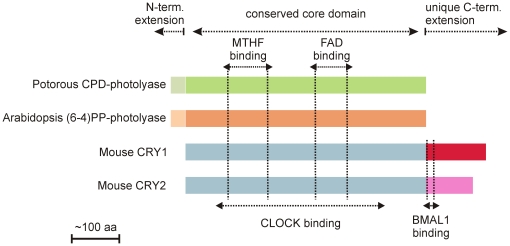
Cryptochromes and photolyases. Schematic representation of *Pt*CPD-PL, *At*(6-4)PP-PL and mouse CRY1 and 2. Conserved and unique domain are indicated above, chromophore binding site are indicated by vertical doted lines. CLOCK and BMAL1 binding regions in mouse CRY and indicated below.

Phylogenetic analysis has shown that the CPF is divided in two major subgroups. The first subgroup encompasses (i) class I CPD photolyases, (ii) (6-4)PP photolyases and animal cryptochromes, (iii) plant cryptochromes, and (iv) DASH cryptochromes, whereas the second subgroup is solely composed of class II CPD photolyases [29]. It is accepted that all members of the photolyase/cryptochrome protein family evolved from a common ancestor CPD photolyase by multiple gene duplications [34]. Cryptochromes and photolyases on the one hand share a common backbone, the core domain, which binds two chromophoric cofactors (i.e. FAD and either 5,10-methenyl-tetrahydrofolate or 8-hydroxy-5-deazaflavin), but on the other hand differ in the presence of N- and C-terminal extensions (see Figure 1). Whereas eukaryotic photolyases have an N-terminal extension, containing nuclear and mitochondrial localization signals, cryptochromes contain a unique C-terminal extension of variable length and amino acid composition. It is currently accepted that the functional diversity among cryptochromes (i.e. photoreceptor, circadian photoreceptor, or core clock protein) is achieved by the diversity of their C-terminal extensions.

Detailed structure/function analysis of the C-terminal region of mammalian CRY1 allowed us to identify a putative coiled-coil domain at the beginning of the C-terminal extension as a potential PER and BMAL1 binding site [35]. Deletion of the complete C-terminal extension (aa 471-606 of mouse CRY1) abolished the CLOCK/BMAL1 transcription inhibitory potential of CRY1, Similarly, Green and co-workers have demonstrated that the C-terminal extension of *Xenopus laevis* CRY proteins is crucial for transcription repression [36]. Interestingly, specific deletion of either the coiled-coil domain (aa 471-493) or the downstream tail region (aa 494-606) of mammalian CRY1 failed to eliminate its ability to inhibit CLOCK/BMAL1-mediated transcription [35], likely because these mutant proteins can still bind to CLOCK via a yet unidentified region of the core domain. This finding lead us to suggest that an interaction between the C-terminal extension and the core domain is mandatory for the clock function of mammalian cryptochromes, possibly by providing structure to the latter [35].

In the present study, we have explored the importance of the core domain of mammalian CRY proteins for core oscillator function by addressing the question to what extent photolyase enzymes affect circadian core oscillator function. Using *in vivo* (photolyase transgenic mice) and *in vitro* (cellular clock reporter and CLOCK/BMAL1 transcription assays) approaches, we show that *Potorous tridactylus* CPD photolyase (hereafter referred to as *Pt*CPD-PL) not only displays cryptochrome-associated functions, but also can replace the CRY proteins in the mammalian circadian core oscillator.

## Materials and Methods

### Ethics statement

Mice were kept at the Animal Resource Center (Erasmus University Medical Center), which operates in compliance with the European guidelines (European Community 1986) and The Netherlands legislation for the protection of animals used for research, including ethical review. Animal studies at Erasmus University Medical Center were approved by DEC Consult, an independent Animal Ethical Committee (Dutch equivalent of the IACUC) under permit numbers 139-09-02 (EUR1702) 139-09-11 (EUR1760) and 139-09-12 (EUR1761).

### Mouse lines and monitoring of circadian behavior

β-actin::*At*(6-4)PP-PL [33], β-actin::*Pt*CPD-PL [31], and *Per2::Luc* transgenic mice (generation described below), as well as *Cry1* ^-/-^/*Cry2* ^-/-^ knockout mice [9], all in a C57BL/6J background, were housed under standard conditions and fed *ad libitum*. All mouse lines were backcrossed at least 11 times to a C57BL/6J background. For the monitoring of locomotor activity rhythms, male mice (12–16 weeks) were individually housed in a light-proof chamber in cages (30×45 cm) equipped with a running wheel (14 cm in diameter) and a sensor system to detect wheel revolutions. Animals were maintained in a cycle of 12 h light (150 lux) and 12 h darkness (LD) or in continuous darkness (DD) in constant ambient temperature with water and food available *ad libitum*. Voluntary wheel running (wheel revolutions per unit of time) was continuously recorded by an online computer using the ERS program. Activity records were plotted as actograms and the period of locomotor activity was determined by the chi-square method. Unpaired Student's t-tests were used to make statistical comparisons between the different genotypes.

### Generation of *Per2::Luc* transgenic mice

The construct used to generate the *Per2::Luc* transgenic mice consists of the luciferase gene under control of the *mPer2* promoter, cloned in pBS (Figure S1). The primers used to amplify the 4.2 kb *mPer2* promoter fragment are indicated in Figure S1. Intronic sequences from the rabbit β-globin locus were included in the expression construct for messenger stability. The expression construct fragment was excised from the plasmid using appropriate restriction enzymes, separated from the vector DNA by agarose gel electrophoresis, isolated from the gel with the GeneClean II kit (Bio101), and further purified using Elutip-D-mini columns (Schleicher and Schuell, Dassel, Germany). The fragment was dissolved in injection buffer (10 mM Tris–HCl pH 7.5, 0.08 mM EDTA) and injected in the pronucleus of fertilized eggs derived from FVB/N intercrosses as described [37]. Animals were backcrossed in a C57BL/6J background. Genotyping was performed by PCR using primers located in the luciferase gene (Figure S1). Annealing was performed at 55°C. DNA derived from transgenic mice rendered a PCR product of 475 bp, whereas no product was detected using DNA from wild type litter mates.

### RNA isolation and quantitative PCR

Coronal cryrosections (25 µm) mounted on 1 mm PALM pen-membraneTM slides were rapidly thawed, fixed for 30 seconds in 70% EtOH and immediately stained with haematoxylin for 3 minutes. Following staining sections were rinsed in DEPC treated dH2O and dehydrated by several rinses in 100% EtOH. Laser catapult microdissection (LCM) of the SCN was accomplished using the PALM Microlaser system on freshly prepared sections. Isolated SCN was dissolved immediately in Lysis Buffer (Qiagen) and stored at −80°C for subsequent RNA purification. RNA was purified with the inclusion of ‘on-column’ DNase treatment using the Qiagen RNeasy ‘Micro’ kit according the manufacturers protocol, except that an additional elution was performed in the final step to maximize RNA yield. RNA eluted with RNase-free dH_2_O was vacuum evaporated for immediate amplification and cDNA generation. Quality of the freshly purified RNA was assayed using the Agilent BioAnalyzer in combination with the RNA ‘Pico’ chip. When intact 18S/28S ribosomal RNA peaks were evident, the sample was considered worthy of assay by Q-PCR.

Amplification was accomplished using the Ovation™ RNA Amplification System V2 according to the manufactures protocols (Nugen Technologies Inc). Efficiency of the amplification was assayed quantitatively by 260/280 nm estimation of cDNA concentration, where a yield of at least 4.8 µg cDNA was deemed sufficient for specific amplification of the RNA template; and qualitatively using the BioAnalyzer with the RNA ‘Nano’ chip to confirm that the majority of unfragmented, amplified cDNA is approximately of 900 bp in length. Quantitative PCR for the determination of *At*(6-4)PP-PL and *Pt*CPD-PL mRNA levels was performed in triplicate using an iCycler iQ™ Real-Time PCR Detection System (BioRad), SYBR-green and primers [31], [33] generating intron-spanning products of 150-300 bp. Expression levels were normalized to *Hprt* (hypoxanthine guanine phosphoribosyl transferase) mRNA levels. The generation of specific products was confirmed by melting curve analysis, and primer pairs were tested with a logarithmic dilution of a cDNA mix to generate a linear standard curve, which was used to calculate primer pair efficiencies.

### Cell culture and transfection

COS7 [35], NIH3T3 (American Type Culture Collection), and HEK293T (American Type Culture Collection) cells, as well as primary wild type and *Pt*CPD-PL mouse dermal fibroblasts (MDFs) and immortalized *Cry1^-/-^/Cry2^-/-^* MDFs, were cultured in Dulbecco's modified Eagle's medium-F10-Pen/Strep-10% fetal calf serum. To generate MDFs, mice were sacrificed by cervical dislocation, and a small piece of back skin of the mouse was removed and cut into pieces with a razor blade). Skin pieces were washed in ethanol, rinsed in phosphate-buffered saline, and incubated overnight in medium supplemented with 1.6 mg/ml collagenase type II. Single cells were obtained by passing through a cell strainer, and collected by centrifugation for 5 min at 500 g, resuspended in culture medium, and seeded onto a 10 cm dish. MDFs were cultured in a low-oxygen incubator (5% CO_2_, 3% O_2_). Transient expression studies were performed by transfecting cells with plasmids using Fugene reagent (Boehringer) according to the manufacturer's instructions. The following pCDNA3-based plasmids (Invitrogen) were used: pcDNA-HA-mCry1, pcDNA-*Pt*CPD-PL, pcDNA-Bmal1 and pcDNA-Clock. pcDNA-*Pt*CPD-PL is based on the construct used to generate the transgenic mice [31]. For luminescence measurements pGl4.11-Bmal1::luciferase (kindly provided by Dr. U. Schibler, Geneva) was used as a reporter.

### Real time bioluminescence monitoring

To monitor circadian oscillations in cultured cells in real time, cells were cultured in medium buffered with 25 mM HEPES and containing 0.1 mM luciferin (Sigma). After synchronization of intracellular clocks by treatment of confluent cultures with forskolin (dissolved in 100% ethanol, added to the culture medium at a final concentration of 30 µM), bioluminescence was recorded for 7 days (75 sec measurements at 10 min intervals) with a LumiCycle 32-channel automated luminometer (Actimetrics) placed in a dry, temperature-controlled incubator at 37°C. Data was analyzed with the Actimetrics software and two sample comparisons were done using a Students T-test. Amplitudes were calculated both with Actimetrics software, on base-line subtracted data, and by comparing peak versus trough values for RAW data. Control amplitude was set at 100%. Both methods were comparable.

### CLOCK/BMAL1 transcription reporter assay

To determine the capacity of *Pt*CPD-PL to inhibit CLOCK/BMAL1 driven transcription, we used a luciferase reporter assay as previously described [12], [35]. COS7 cells were transfected with 200 ng of the *mPer1*::*luciferase* reporter construct and 15 ng of null-*Renilla luc*, which was used as an internal control. Clock, Bmal1, Cry1 and *Pt*CPD-PL plasmids were added as indicated in the figure legend. The total amount of DNA transfected was kept constant at 2 µg by supplementing with empty pcDNA3.1 vector (Invitrogen). Transcriptional activity was assessed with the Dual-Luciferase 10 Reporter Assay System (Promega) by measuring a ratio of firefly luciferase activity to *Renilla* luciferase activity in each cellular lysate.

### Co-immunoprecipitation experiments

Co-immunoprecipitation studies were performed as described previously [21]. In short, we transiently expressed a *Pt*CPD-PL and either Flag-Bmal1 and/or Flag-Clock in HEK 293T cells and used anti-FLAG antibodies (Sigma) and anti-*Pt*CPD-PL [31] antibodies for the immunoprecipitation and immunoblot analysis step (1∶1000 dilution). As secondary antibody, we used horseradish peroxidase conjugated anti-mouse IgG (DAKO) and anti-rabbit IgG (BioSource) at a 1∶1000 dilution. Chemoluminescence was detected using the ECL system (Pharmacia Biotech).

### Hydroporation experiments

Hydrodynamic tail vein injection experiments were performed as described [38]. In brief, a sterile Ringers Solution (0.9% NaCl, 0.3% KCl, 0.13% CaCl_2_) containing a total of 10 µg plasmid DNA was rapidly injected (8–10 sec) in the tail vein of the mouse under isoflurane anesthesia. This induces uptake of DNA by the liver, which is initially transient and a small proportion will integrate upon regeneration of the liver. Expression of the hydroporated constructs was non-invasively analyzed using an IVIS® Spectrum imaging device (Caliper/Xenogen) (Figure S2), and positive mice were selected for liver isolation and slicing. Animals were sacrificed 24 h after injection (transient expression) and the livers were rapidly removed and placed in ice cold Hank's balanced salt solution supplemented with 50 mM glucose, 4 mM sodium bicarbonate, 10 mM HEPES, 10.000 unit/ml penicillin/10.000 µg/ml streptomycin [5]. Liver slices (200 µm) were prepared using an automated Krumdieck tissue slicer (Alabama R&D). Individual slices were placed on a membrane insert (Millipore) in a 35-mm dish in imaging medium (DMEM supplemented with 0.1 mM luciferin, 2% B27 supplement, 4 mM sodium bicarbonate, 10 mM HEPES, 2.5 ml 10.000 units/ml penicillin/10.000 µg/ml streptomycin). Real time imaging and synchronization were performed as described above. The plasmids used were pGl4.11-Bmal1::luciferase and pcCry1::PtCPD-PL. Cloning and characterization of the *Cry1* promoter will be described elsewhere (Saito and van der Horst, unpublished data).

## Results

### *Potorous tridactylus* CPD photolyase transgenic mice have a short period circadian clock

**Figure 2:**
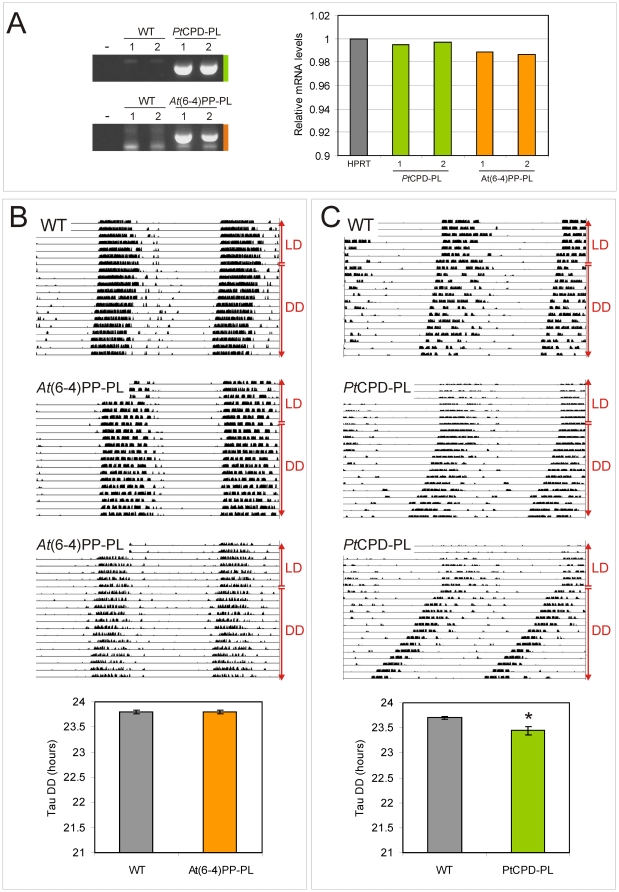
Circadian behavior of photolyase transgenic mice. (**A**) Quantitative RT-PCR analysis of photolyase mRNA levels in the laser-microdissected SCN of *Pt*CPD-PL and *At*(6-4)PP-PL transgenic mice. Left panel: Ethidium bromide stained gel of PCR amplified cDNA, obtained from two independent transgenic mice and corresponding wild type littermates (sacrificed at ZT3). Right panel: graphic representation of quantitative RT-PCR amplification data from two independent animals per genotype (see Experimental procedures for details). The Y-axis represents the *Pt*CPD-PL and *At*(6-4)PP-PL mRNA levels relative to that of *Hprt*. (**B, C**) Circadian behavior of *At*(6-4)PP-PL (B) and *Pt*CPD-PL (C) transgenic mice and corresponding littermates (n  =  10 per genotype). Animals were kept under normal light conditions (LD 12∶12 h) and subsequently exposed to constant darkness (DD) (indicated on the right side of the panels). Shown are representative examples of double-plotted actograms and graphic representations of the free-running period (τ) in constant darkness (bottom panels). Error bars represent the standard error of the mean (SEM); the asterisk indicates significance (p = 0.03).

To investigate whether their strong structural resemblance to cryptochromes (see Figure 1) allows photolyases to interfere with mammalian circadian core oscillator function, we took advantage of the availability of β-actin promoter-driven *Potorous tridactylus* CPD photolyase and *Arabidopsis thaliana* (6-4)PP photolyase transgenic mice (hereafter referred to as *Pt*CPD-PL and *At*(6-4)PP-PL mice), previously generated in our laboratory. These animals carry 3 copies of the *Pt*CPD-PL and *At*(6-4)PP-PL transgene, respectively, and express an active photolyase, capable of removing DNA lesions from the DNA in a light-dependent manner [31], [33]. As shown in Figure 2A, and as could be expected on the basis of the ubiquitous expression of the *β-actin* promoter, quantitative RT-PCR analysis of mRNA derived from laser microdissected SCN revealed that *Pt*CPD-PL and *At*(6-4)PP-PL mice express the photolyase transgene in the SCN at comparable levels to the *Hprt* gene.

We next addressed the question whether expression of photolyase in the SCN would affect the circadian behavior of the mouse. To this end, we measured circadian wheel-running behavior of photolyase transgenic mice and sex and age-matched control littermates under normal light/dark (LD) cycles and in constant darkness (dark/dark; DD). As shown in Figure 2B, the period length (tau or τ) of circadian behavior of *At*(6-4)PP-PL transgenic mice is indistinguishable from that of the corresponding wild type littermates. In marked contrast, *Pt*CPD-PL transgenic mice revealed a small but significant shortening of the period length of (15 to 20 min, p<0.05), as compared to wild type littermates (Figure 2C). In addition, the tau of the *Pt*CPD-PL mice has a larger (2-fold) variation than that of control littermates, possibly derived from small individual differences in expression levels.

These findings strongly suggest that expression of *Potorous tridactylus* CPD photolyase in the SCN interferes with circadian clock performance. In contrast, *Arabidopsis thaliana* (6-4)PP photolyase does not appear to influence the circadian clock. This observation is in agreement with our finding that *At*(6-4)PP-PL is not able to inhibit CLOCK/BMAL1 [35]. We therefore further will focus on the effect of *Pt*CPD-PL on circadian rhythms.

### *Potorous tridactylus* CPD photolyase dampens the circadian core oscillator

**Figure 3:**
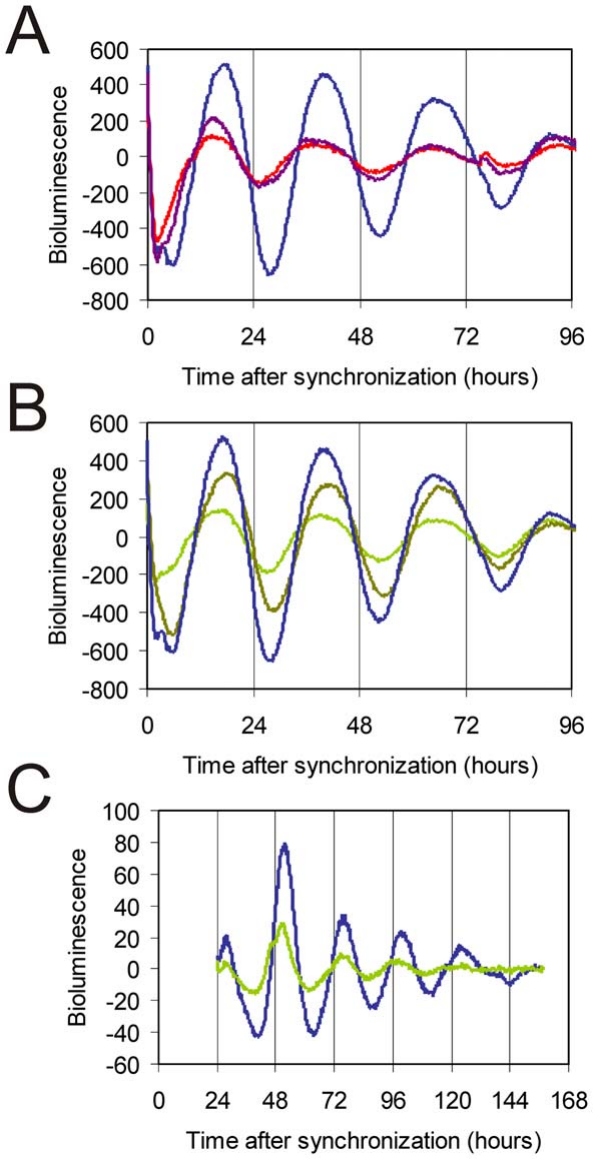
*Pt*CPD photolyase dampens circadian oscillations. (**A, B**) Representative examples of bioluminescence rhythms in NIH3T3 cells co-transfected with a mBmal1::luciferase reporter construct and either empty pcDNA3 (blue line), pcDNA-Cry1, (100 ng, purple line; 200 ng, red line) or pcDNA-CPD photolyase (200 ng, olive line; 400 ng, green line). Empty pcDNA3 vector was added to correct for the amount of DNA transfected. (**C**) Representative example of bioluminescence rhythms in primary MDFs, derived from *Pt*CPD photolyase transgenic mice (green line) and wild type littermates (blue line), transiently expressing the Bmal1::luciferase reporter gene. Bioluminescence recordings were started immediately after forskolin synchronization of the individual cellular clocks. The Y-axis represents base line subtracted bioluminescence values.

As photolyases structurally resemble cryptochromes [29], [34], and given the observation that transient constitutive overexpression of the CRY1 protein suppresses the rhythmic expression of a cotransfected Bmal1::Luc reporter gene (Figure 3A), we next investigated the effect of transient overexpression of *Pt*CPD-PL on the circadian clock of cultured fibroblasts. After synchronization of the individual intracellular circadian clocks with forskolin [39], cells cotransfected with the Bmal1::Luc reporter construct and empty pcDNA3 vector (used as a negative control) were shown to oscillate with a period of 25.6±0.2 hr (n = 11). Interestingly, overexpression of *Pt*CPD-PL reduces the amplitude of the oscillations in a dose-dependent manner (Figure 3B; Table 1).

**Table 1:** 

NIH 3T3 cells		
	*Tau (hours)*	*Amplitude (%)*
*control*	25.60±0.16	100
*PtCPD-PL*	25.57±0.16	65±7

MDF lines		
	*Tau (hours)*	*Amplitude (%)*
*WT*	23.83±0.18	100
*PtCPD-PL*	22.70±0.43	62±5

To study the influence of CPD photolyase on core oscillator performance under physiological conditions, *Pt*CPD-PL mice were interbred with *Per2::Luc* mice to obtain primary CPD-photolyase mouse dermal fibroblast (MDF) lines containing a clock reporter. Bioluminescence rhythms in *Pt*CPD-PL/Per2::Luc MDFs show a reduction in amplitude (38±5%) when compared to those in Per2::luc (control) fibroblasts (Figure 3C; Table 1), thus confirming the data obtained in the transient expression studies. Interestingly, and in line with the animal studies, the period of oscillations in *Pt*CPD-PL/Per2::Luc MDFs (22.7±0.4 hr; n = 4) is approximately 50 min shorter (p<0.05) than that of Per2:Lluc fibroblasts that do not carry the CPD photolyase transgene (23.8±0.2 hr; n = 4).

Taken together, these data demonstrate that the *Pt*CPD-PL exerts a dominant negative effect on the circadian clock by dampening the oscillations and shortening the period length, likely by interfering with CRY mediated functions.

### *Potorous tridactylus* CPD photolyase inhibits CLOCK/BMAL1-driven transcription

**Figure 4:**
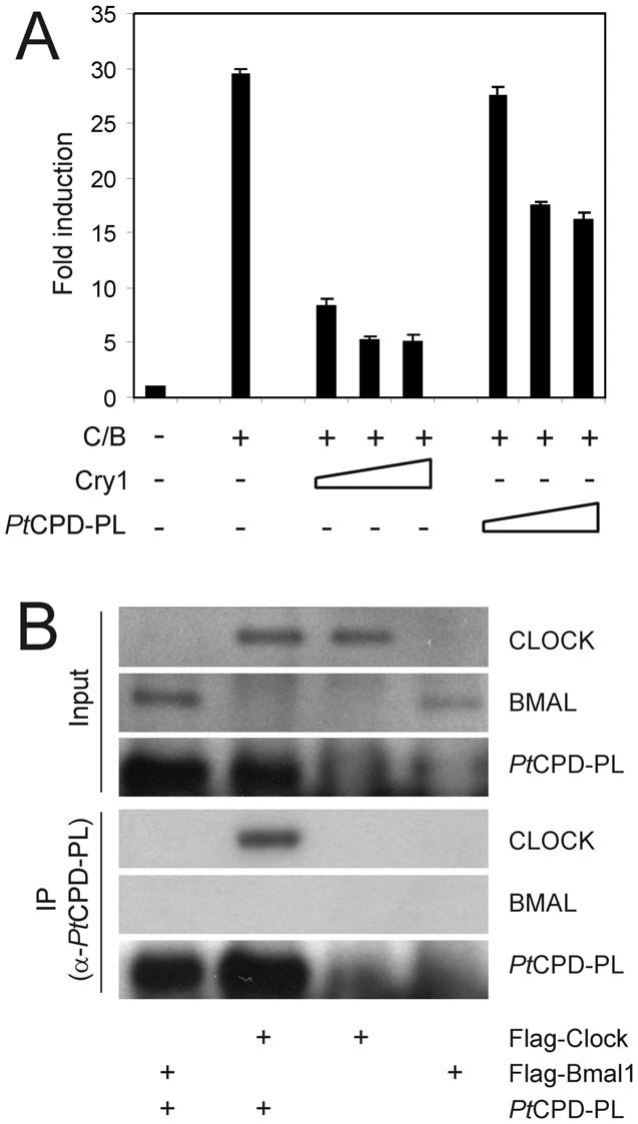
*Pt*CPD photolyase represses CLOCK/BMAL1-driven transcription and interacts with CLOCK. (**A**) COS7 cell-based CLOCK/BMAL1 transcription assay using a *mPer1* E-box promoter-luciferase reporter construct. Luminescence, shown as x-fold induction from the basal expression level (set to 1), is indicated on the Y axis. pcDNA3, pRL-CMV, and the mPer2::luc were added in all reactions. The presence or absence of Cry1 (10-100 ng) and *Pt*CPD-PL (100-300 ng) expression plasmids is indicated below the graph. Empty pcDNA3 vector was added to correct for the amount of DNA transfected. Mean and standard deviation of triplicate samples are shown. (**B**) Identification of photolyase-binding proteins. *Pt*CPD-PL was precipitated from HEK293T cells, transfected with *Pt*CPD-PL, Flag-Clock or Flag-Bmal1 or double transfected with *Pt*CPD-PL and either Flag-Clock or Flag-Bmal1. Upper panels: Immunoblot analysis of total cell lysates, confirming the presence of the various transiently expressed proteins. Lower panels (IP): Immunoblot analysis of precipitated *Pt*CPD-PL (anti-*Pt*CPD-PL antibodies) and CLOCK and BMAL1 (anti-FLAG antibodies).

The dominant negative effect of CPD photolyase on cellular clock performance and circadian behavior, as evident from the *in vivo* and *in vitro* studies, prompted us to investigate the underlying mode of action. Since CRY proteins are strong inhibitors of the CLOCK/BMAL1 transcription activator [12], we used a COS7 cell based reporter assay to analyze the ability of *Pt*CPD-PL to inhibit CLOCK/BMAL1-driven transcription of the *mPer1* promoter-driven luciferase reporter gene. Consistent with previous studies [12], [35] simultaneous expression of CLOCK and BMAL1 causes a 30-fold induction in transcription of the luciferase gene, which is strongly repressed in the presence of CRY1 (Figure 4A). Interestingly, notwithstanding the fact that the protein should be expressed at high level, the *Pt*CPD-PL is also capable of significantly suppressing CLOCK/BMAL1 activity.

From these data we conclude that *Pt*CPD-PL is able to interfere with mammalian core oscillator performance by exerting a cryptochrome-like function, i.e. inhibition of CLOCK/ BMAL1-mediated transcription.

### *Potorous tridactylus* CPD photolyase interacts with CLOCK

We have previously shown that the inhibition of CLOCK/BMAL1 mediated transcription by CRY1 requires a complex network of interactions with CLOCK and BMAL1, involving the CRY1 core domain and C-terminal extension [35] (see also Figure 1). We therefore next asked the question whether *Pt*CPD-PL, like CRY proteins, can physically interact with CLOCK and BMAL1. To this end, we performed a co-immunoprecipitation experiment using HEK293T cells overexpressing the FLAG-CLOCK or FLAG-BMAL1 proteins alone, or in combination with *Pt*CPD-PL (Figure 4B). In the absence of *Pt*CPD-PL neither CLOCK nor BMAL1 were pulled down with anti-*Pt*CPD-PL antibodies, which excludes non-specific binding of the antibodies to these proteins. However, in the presence of *Pt*CPD-PL, the CLOCK protein is shown to co-precipitate with *Pt*CPD-PL, whereas the BMAL1 protein is not. This result indicates that *Pt*CPD photolyase inhibits CLOCK/BMAL1-mediated transcription through direct interaction with CLOCK.

### *Potorous tridactylus* CPD photolyase can replace cryptochromes in the mammalian circadian oscillator

**Figure 5:**
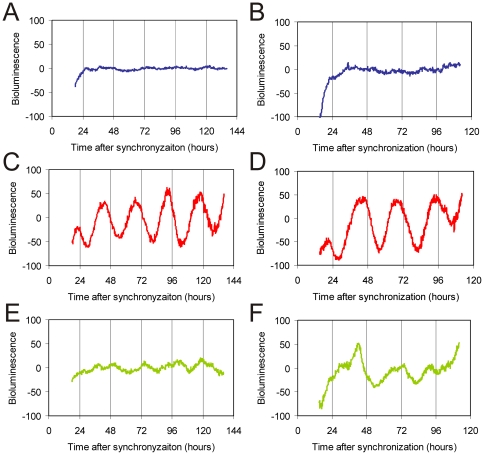
Correction of the circadian clock in fibroblast lines derived from CRY-deficient mice. Representative examples of bioluminescence rhythms in immortalized MDF lines, derived from *Cry1^-/-^/Cry2^-/-^* mice, and stably expressing either empty pcDNA3 (**A, B**), pcCry1::Cry1 (**C, D**) or pcCry1::*Pt*CPD-PL (**E, F**), and transfected with the reporter construct. Y-axis represents base line subtracted bioluminescence values.

Having shown that *Pt*CPD-PL can interact with CLOCK, leading to inhibition of CLOCK/BMAL1-driven transcription, the intriguing question arises whether this photolyase can actually replace the CRY proteins in the mammalian circadian oscillator and rescue the arrhythmicity of *Cry1^-/-^/Cry2^-/-^* mice. Generation of a new *Cry1* promoter-driven *Pt*CPD-PL transgenic mouse line in a *Cry*-deficient background and subsequent behavioral analysis would be extremely time-consuming. As peripheral circadian clocks form a good model for the master clock in the SCN [40], [41], we chose to generate stable fibroblast lines, derived from *Cry1^-/-^/Cry2^-/-^* mice, transfected with pCry1::*Pt*CPD-PL, pCry1::Cry1 or the empty vector. These cells were then transiently transfected with Bmal1::Luc reporter construct, synchronized with forskolin, and subjected to real time luminescence monitoring. As expected, expression of pCry1::Cry1 (C, D) restores circadian rhythms in arrhythmic *Cry1^-/-^/Cry2^-/-^* fibroblasts (Figure 5C and D) , whereas the empty vector has no effect (Figure 5A and B). Interestingly, pCry1::*Pt*CPD-PLinduces oscillations (Figure 5E and F). Although the period appears in the circadian range, the irregular character of the oscillations precluded calculation of tau.

**Figure 6:**
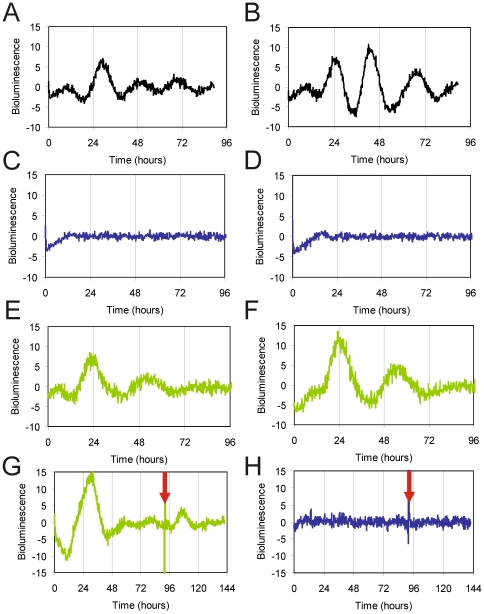
*Pt*CPD photolyase corrects the circadian clock in the liver of CRY-deficient mice. Representative examples of bioluminescence rhythms in liver slices obtained from mice hydroporated with pGL4.11-Bmal1::Luc (black line), together with either empty pcDNA3 (blue line) or pcDNA-CPD photolyase constructs (green line). (**A, B** )Liver slices from control mice, injected with the reporter construct only. (**C-H**) Liver slices from *Cry1^-/-^/Cry2^-/-^* mice injected with either empty pcDNA3 (C, D, H) or pcCry1::*Pt*CPD-PL (E-G), in addition to the reporter construct. In some experiments (G and H) the slices were treated with Forskolin at 96 h to resynchronize the circadian clock. The Y-axis represents base line subtracted bioluminescence values.

Taking into account the irregular character of the above mentioned oscillations, we took an alternative approach to further to study the capacity of *Pt*CPD-PL to rescue circadian rhythms in tissues. To this end, we used the hydroporation technique [38] to introduce clock reporter and CPD photolyase expression constructs in the mouse liver. Twenty-four hours after co-injection of pGL4.11-Bmal1::Luc and either pCry1::*Pt*CPD-PL or the empty vector in the tail vein of the mouse, liver slices were prepared and clock performance was monitored by real-time imaging of bioluminescence. Co-injection of the Bmal1::Luc reporter plasmid and empty vector in *Cry1^-/-^/Cry2^-/-^* mice and *Cry1^+/-^/Cry2^+/-^* littermate controls resulted in rhythmic expression of the luciferase reporter gene in *Cry1^+/-^/Cry2^+/-^* liver slices (Figure 6A and B), whereas, in line with the absence of a circadian clock, bioluminescence levels remained flat in liver slices from *Cry1^-/-^/Cry2^-/-^* mice (Figure 6C and D), thus validating the hydroporation approach. Interestingly, upon co-injection of the Bmal1::Luc reporter plasmid and pCry1::*Pt*CPD-PL and *Pt*CPD-PL expression construct in *Cry1^-/-^/Cry2^-/-^* animals, we observed a reinitiation of circadian rhythmicity in *Cry*-deficient liver slices for at least two cycles (Figure 6E and F). This oscillation dampened rapidly, but could be revived for at least one cycle by forskolin treatment of the liver slices (Figure 6G). As in the absence of *Pt*CPD-PL forskolin did not exert any effect on bioluminescence levels in *Cry1^-/-^/Cry2^-/-^* slices (Figure 6H), the forskolin-induced bioluminescence rhythm in *Pt*CPD-PL expressing *Cry1^-/-^/Cry2^-/-^* slices can only be explained by resynchronization of (running) intracellular clocks.

From these data we conclude that rhythmically expressed *Pt*CPD photolyase can functionally substitute for CRY proteins in the mammalian circadian oscillator and that such a *Pt*CPD-PL-driven molecular oscillator can still respond to non-photic clock-synchronizing stimuli (i.e. forskolin).

## Discussion

In the present study, we analyzed the capacity of two different photolyases to interfere with circadian clock performance: the Class II CPD photolyase from *Potorous tridactylus* (*Pt*CPD-PL), which is only distantly related to CRY1, and the (6-4)PP photolyase from *Arabidopsis thaliana* (*At*(6-4)PP-PL), which is closely related to CRY1. In line with our observation that *At*(6-4)PP-PL does not inhibit CLOCK/BMAL1 transcriptional activity [35], analysis of the circadian behavior of *β-actin* promoter-driven *At*(6-4)PP-PL transgenic mice (ubiquitously expressing the transgene, including the SCN) did not reveal any dominant negative effect of the photolyase on circadian period length. In marked contrast, *β-actin* promoter-driven *Pt*CPD-PL transgenic mice showed a small but significant reduction of the period length of circadian behavior. In support of this *in vivo* observation, we also obtained a shortening of the period length of the molecular oscillator in cultured *Pt*CPD-PL dermal fibroblasts. This accelerated clock in *Pt*CPD-PL transgenic mice is unlikely an artifact resulting from unintended inactivation of known (core) clock genes, as analysis of the sequences flanking the integration site of the transgene excluded the presence of such genes (data not shown). Moreover, transiently overexpressed *Pt*CPD-PL was able to dampen the circadian oscillator in NIH3T3 cells. In this respect PtCPD-PL resembles CRY1, which when overexpressed also blunts circadian rhythms. The effect of *Pt*CPD-PL on circadian rhythms is mediated by repression of CLOCK/BMAL1-mediated transcription via direct physical interaction with CLOCK, but not with BMAL1.

Previously, we have shown that removal of the complete C-terminal extension of CRY1 abolishes repressor activity towards CLOCK/BMAL1 driven transcription of E-box containing clock genes and clock-controlled genes [35]. In the same study, we demonstrated that whereas *At*(6-4)PP-PL by itself has no effect on CLOCK/BMAL1, fusion of the last 100 aa of the CRY1 core domain in conjunction with its C-terminal extension (aa 371-606) to *At*(6-4)PP-PL) resulted in a chimeric protein which is still able to inhibit CLOCK/BMAL1-mediated transcription. Based on these findings, we hypothesized that acquirement of C-terminal extensions (to the core domain) during evolution functionally separated cryptochromes from photolyase and conferred a clock function to the CRY proteins [35]. We now provide evidence that *Pt*CPD-PL harbors core clock features that allow it to repress CLOCK/BMAL1 transcriptional activity and function as a true cryptochrome. Considering that *At*(6-4)PP-PL is more homologous to CRY1 than *Pt*CPD-PL, our findings suggest that it is not the primary amino acid sequence per sé, but rather the overall structure of the core domain, that makes a photolyase repressing CLOCK/BMAL1-mediated transcription. Our results indicate that the *Pt*CPD-PL by itself has the proper structure, whereas *At*(6-4)PP-PL gains such a structure after fusion with a C-terminal extension of mammalian CRY1 [35]. In addition, as *Pt*CPD-PL fails to bind BMAL1, interaction with CLOCK is sufficient to inhibit CLOCK-BMAL1-mediated transcription, which is in complete agreement with our previous observation that the BMAL1-binding coiled-coil domain in the C-terminal extension of CRY1 can be deleted without major consequences [35].

Given the dominant negative effect of constitutive *Pt*CPD-PL expression on circadian behavior of the mouse (*in vivo* data), it is tempting to speculate on the underlying molecular mechanism by which *Pt*CPD-PL can inhibit CLOCK/BMAL1-driven transcription (*in vitro* data) and its impact on circadian core oscillator performance. By interacting with CLOCK, *Pt*CPD-PL may prevent the formation of CLOCK/BMAL1 heterodimers and/or binding of the CLOCK/BMAL1 heterodimer to E-box promoters in the DNA. In this scenario, the photolyase reduces the efficiency at which E-box containing clock (controlled) genes are transcribed by reducing the number of available transcription activators. However, as we have shown that a *Chrysodeixis chalcites* nucleopolyhedroviral photolyase can bind to mammalian CLOCK without affecting CLOCK/BMAL1 transcription potential, binding of a CPF protein per se does not prevent CLOCK/BMAL1 heterodimerization and DNA binding (Biernat and Chaves, submitted for publication). Therefore, a more plausible explanation would be that binding of *Pt*CPD-PL to CLOCK inhibits transcription activation of E-box promoter-bound CLOCK/BMAL1 heterodimers in a cryptochrome like manner. Strikingly, using an *in vivo* hydroporation approach, we show that *Pt*CPD-PL can rescue the lost circadian oscillator in CRY-deficient cells. When expressed from the *Cry1* promoter, *Pt*CPD-PL revived rhythmic expression of the *Bmal1::luciferase* reporter gene in the *Cry1^-/-^/Cry2^-/-^* mouse liver explants. Moreover, the period of oscillations was in the same range as that of a CRY-driven oscillator and responds to non-photic phase synchronizing stimuli (i.e. forskolin). We therefore conclude that the *Pt*CPD-PL protein has the potential to act as a true mammalian CRY protein.

While this work was in progress, two other members of the CPF have been shown to maintain dual functions: the *Pt*CPF1 protein from the marine diatom *Phaeodactylum tricornutum* and the *Ot*CPF1 protein from the green algae *Ostreococcus tauri.* These proteins hold (6-4)PP photolyase activity and (like *Potorous* CPD-PL) can inhibit CLOCK/BMAL1 driven transcription in a heterologous mammalian system [42], [43] and are therefore considered a missing link in evolution. Interestingly, we found that *Arabidopsis thaliana* (6-4)PP photolyase does not inhibit CLOCK/BMAL1 [35] or affect circadian behavior, whereas the distantly related *Potorous* CPD photolyase does. Moreover, we show that this marsupial class II CPD-photolyase can actually substitute for CRY proteins in the mammalian circadian oscillator. In a parallel study, we have shown that the *Chrysodeixis chalcites* nucleopolyhedrovirus PHR2 protein, another class II CPD photolyase, is able to interact with CLOCK and affect circadian rhythms *in vitro* (Biernat and Chaves, unpublished data).

These findings contribute to understanding the functional evolution of cryptochromes and photolyases. So far, the identified CPF members with a dual function are either (6-4)PP photolyases from lower eukaryotes [42], [43] or class II CPD photolyases (this manuscript; Biernat and Chaves, submitted for publication). Ancestral CPF members were likely proteins with both DNA repair and circadian clock function. We propose that after the divergence of classes I and II, class II CPD photolyases have kept this dual function throughout evolution. Class I CPD photolyases and (6-4)PP photolyases, however, have lost the circadian function in time, which was taken by cryptochromes. In view of this hypothesis, it will be challenging to study molecular clocks in organisms that have both a photolyase with a dual function and cryptochromes, as is the case of marsupials, such as *Potorous* and *Monodelphis*. The genome of *Monodelphis domesticus* has been sequenced and reveals the presence of cryptochrome genes, as well as photolyase. It will be of interest to determine how the clock of non-placental mammals will respond to the loss of photolyase. On the basis of our data, one would predict a change in tau, suggesting that the circadian clock of placental mammals has adapted to the loss of photolyase by adjusting period. Studying the marsupial circadian system at the cellular and molecular level will answer these questions and shed light on the functional evolution of the CPF.

The present study has identified the *Potorous tridactylus* CPD photolyase as an attractive candidate for further structure-function studies, aiming at understanding the functional diversity between cryptochromes and photolyases. Analogous to our previous study with mammalian CRY1 - *Arabidopsis* (6-4)PP photolyase chimeric proteins, it will be informative to swap domains between *Potorous* CPD photolyase and CRY proteins for functional mapping. Such studies will ultimately reveal how nature uses the same core sequence for completely different functions (e.g photoreactivation by photolyases vs clock function of cryptochromes).

## Supporting Information

Figure S1 **Schematic representation of the mPer2::Luc construct used to generate transgenic clock reporter mice.** (**A**) The luciferase gene is cloned in front of the *mPer2 promoter,* using pBS as backbone. Intronic sequences from the rabbit *β-globin* locus were included in the expression construct for messenger stability. Restriction enzyme sites are indicated. (**B**) Sequence of the primers used to amplify the 4.2 kb *mPer2* promoter fragment. (**C**) Sequence of the luciferase primers used to genotype mPer2::Luc mice.
(TIF)

Figure S2 **Detection of luminescence in the liver of hydroporated mice.** Representative examples of dorsal luminescence images, obtained 24 hour after hydroporation of mice with either the Bmal1::Luc reporter construct (left) or the empty vector (right). Expression of the hydroporated constructs was non-invasively monitored in isoflurane anesthetized animals using an IVIS® Spectrum imaging device (Caliper/Xenogen). Colors indicate signal intensity. Note that the reporter is prominently expressed in the liver.
(TIF)
