# Supplementary figures and images for: The Potorous CPD Photolyase Rescues a Cryptochrome-Deficient Mammalian Circadian Clock
Source: PLoS One. 2011 Aug 16;6(8):e23447. doi: 10.1371/journal.pone.0023447 (PMC3156801; doi:10.1371/journal.pone.0023447)

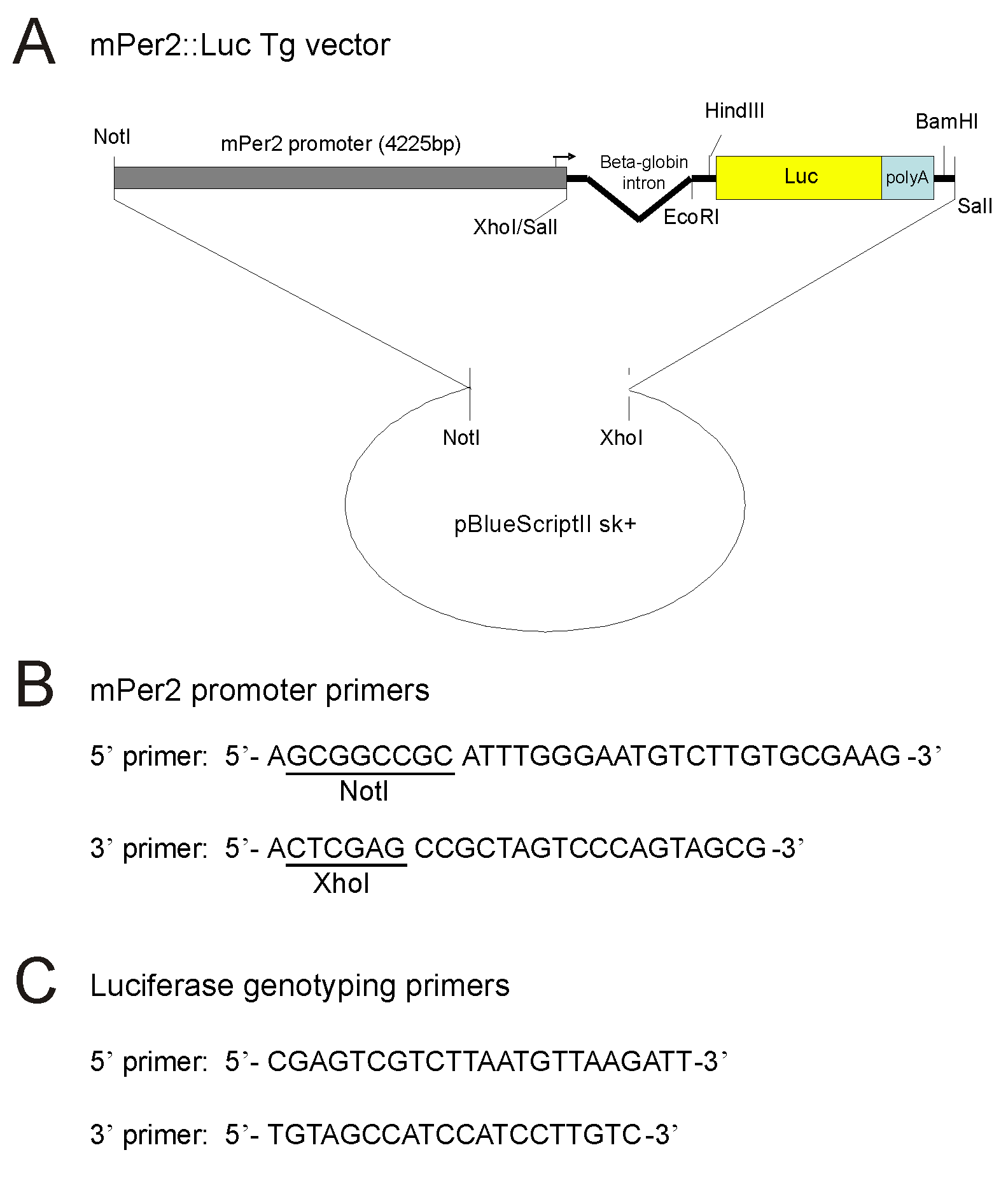

Supplement: Figure S1 — Schematic representation of the mPer2::Luc construct used to generate transgenic clock reporter mice. (A) The luciferase gene is cloned in front of the mPer2 promoter, using pBS as backbone. Intronic sequences from the rabbit β-globin locus were included in the expression construct for messenger stability. Restriction enzyme sites are indicated. (B) Sequence of the primers used to amplify the 4.2 kb mPer2 promoter fragment. (C) Sequence of the luciferase primers used to genotype mPer2::Luc mice. (TIF) [file pone.0023447.s001.tif]

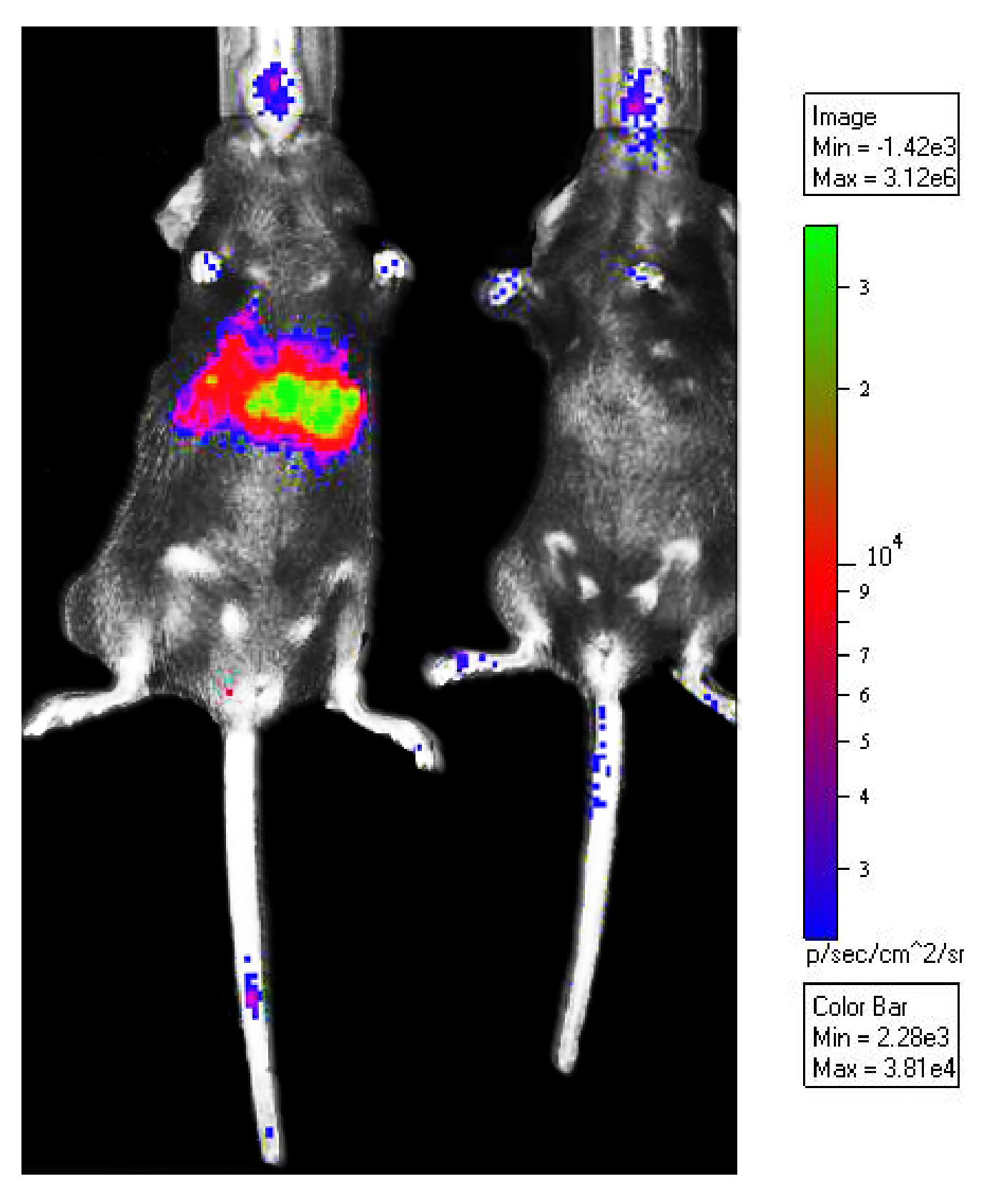

Supplement: Figure S2 — Detection of luminescence in the liver of hydroporated mice. Representative examples of dorsal luminescence images, obtained 24 hour after hydroporation of mice with either the Bmal1::Luc reporter construct (left) or the empty vector (right). Expression of the hydroporated constructs was non-invasively monitored in isoflurane anesthetized animals using an IVIS® Spectrum imaging device (Caliper/Xenogen). Colors indicate signal intensity. Note that the reporter is prominently expressed in the liver. (TIF) [file pone.0023447.s002.tif]
